# Routine Histopathological Evaluation After Appendectomy: Is It Necessary? A Systematic Review

**DOI:** 10.7759/cureus.9830

**Published:** 2020-08-18

**Authors:** Kashish Malhotra, Ashvind Bawa

**Affiliations:** 1 General Surgery, Dayanand Medical College and Hospital, Ludhiana, IND

**Keywords:** appendectomy, appendicitis

## Abstract

Acute appendicitis is one of the most common reasons for acute abdominal pain. Fecaliths and lymphoid hyperplasia are the usual etiology of acute appendicitis, however, other unusual causes can also not be neglected which can be parasitic infections, benign or malignant lesions. Due to substantial lab costs and limited resources, the policy of routine histopathological examination (HPE) of appendectomy samples is being questioned.

PubMed, PubMed Central (PMC), and Google Scholar were used to look for relevant published studies. The following keywords were used both alone and in combination: “Acute appendicitis” and “routine histopathological examination”. Fifteen articles were selected for final review that collectively had 57,524 cases. All these studies included in this systematic review are peer-reviewed.

Based on the reviewed articles, it was found that though the probability of unusual findings in a patient of acute appendicitis is less but it is still significant and if found, often results in a change of management plan of the patient.

Therefore, it is recommended to perform a routine histopathological examination of all appendectomy specimens to rule out unusual pathologies.

## Introduction and background

Acute appendicitis is referred to as inflammation of the inner lining of the appendix, which then may advance to other parts of the organ and surrounding areas. Obstruction of the lumen is one of the major causative factors behind this pathology which can be due to a variety of reasons. Fecaliths and lymphoid hyperplasia are the usual etiology of this clinico-pathological condition, however, other unusual causes can also not be neglected which can be benign neuroma, mucocele, mucinous cystadenoma, endometriosis, gastrointestinal stromal tumor, carcinoid tumors of the appendix, hyperplastic polyp, lymphoma, leukemia, diverticulitis, granulomatous diseases, or infections say amebiasis, enterobiasis, actinomycosis, balantidiasis, schistosomiasis, trichuriasis, or tuberculosis (TB) [1].

Acute appendicitis is one of the most common reasons for acute abdominal pain. Though it can occur in any age group, it is quite frequently seen between the ages of 10 and 20 years. The lifetime risk for males is 8.6% and for females is 6.7% in the United States [2].

The clinical features which primarily put suspicion at diagnosis of acute appendicitis in adults are right lower quadrant pain, abdominal rigidity, and radiation of periumbilical pain to the right lower quadrant. In children, absent or decreased bowel sounds, a positive Psoas sign (pain on passive extension of the right thigh), a positive Rovsing sign (palpation of the left lower quadrant of abdomen intensifies the pain felt in the right lower quadrant), positive Obturator sign (pain on passive internal rotation of the flexed thigh) help in ruling in the diagnosis in favor of acute appendicitis [3].

Surgical appendectomy is the mainstay treatment of acute appendicitis which can be performed either as an open surgery or laparoscopic (minimally invasive) procedure. A recent meta-analysis of randomized controlled trials comparing postoperative results of laparoscopic and open surgery showed that there is lesser postoperative complications, lower incidence of wound infection, shorter duration of stay, and a comparatively faster return to normal activity in favor of laparoscopic operation and a smaller operation time in favor of open surgery via a limited right lower quadrant incision [4].

The protocol of sending appendectomy specimens for histopathological examination (HPE) is variable in various hospitals. This article presents a systematic review from various studies to provide a better basis for assessing the frequency of unusual findings in appendectomy specimens and whether a routine HPE of appendectomy specimens is needed.

## Review

Materials and methods

On the basis of Preferred Reporting Items for Systematic Reviews and Meta-Analyses (PRISMA) guidelines [5], a thorough literature search was conducted using PubMed, PubMed Central (PMC), and Google Scholar for the relevant published studies using the following keywords both alone and in combination: “Acute appendicitis” and “routine histopathological examination”.

Inclusion/Exclusion Criteria

**Figure 1:**
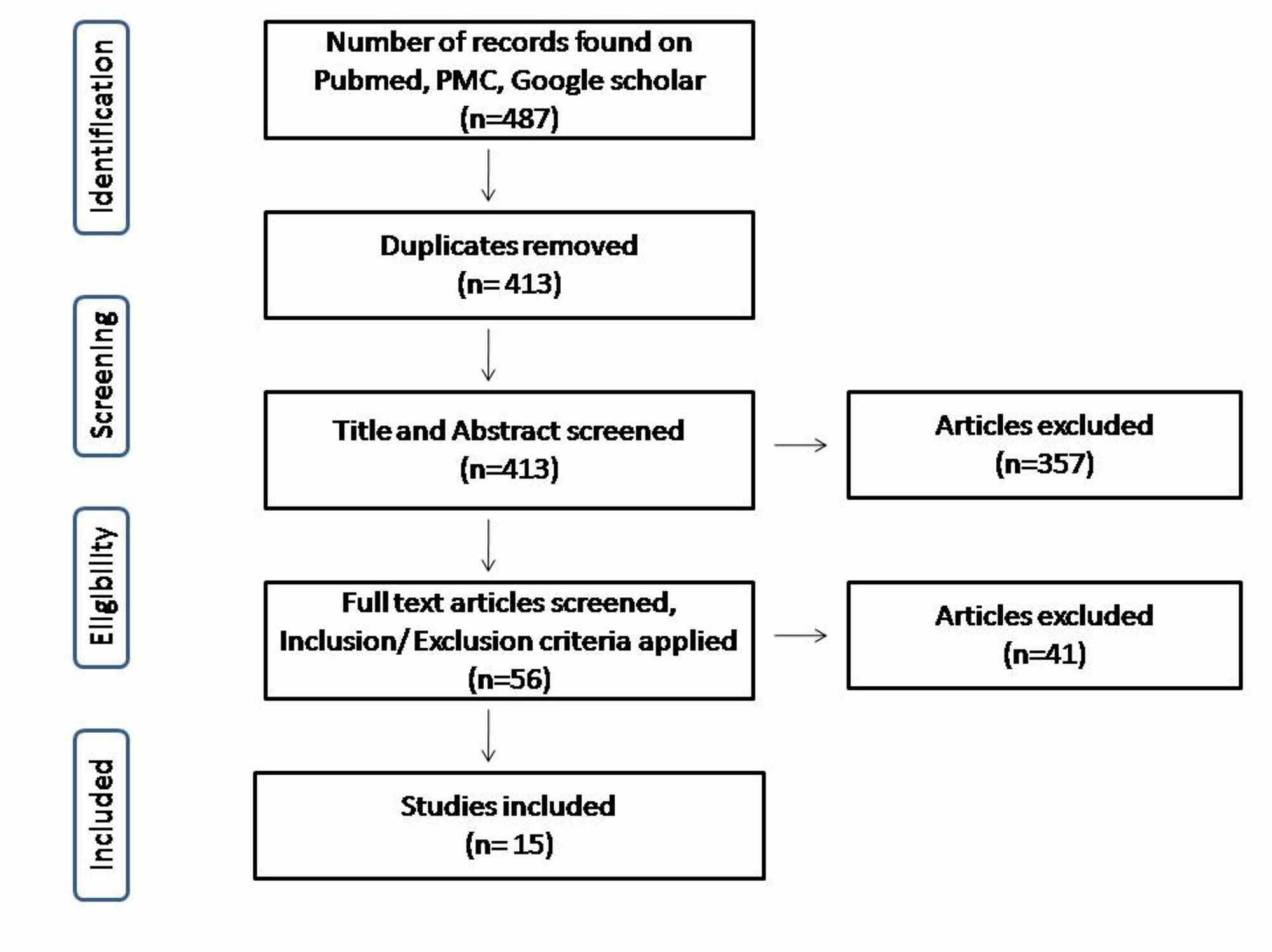
PRISMA diagram showing selection of data

Papers from 2000 till June 2020 in the English language were screened. Research papers in other languages and duplicate papers were not included. Only peer-reviewed articles were included and grey literature was not included. PRISMA diagram depicting the selection and screening of records is shown in Figure 1.

Results

After applying the above-mentioned search criteria, 15 articles were selected for the final review that collectively had 57,524 cases. All these studies included in this systematic review are peer-reviewed.

Details of the studies and relevant related details are summarized in Table 1.

**Table 1:** Data from the studies with basic information regarding the number of patients, purpose and conclusion HPE: Histopathological examination

Author name and year of publication	Study type	No. of patients	Study purpose	Result/Conclusion
Elfaedy et al., 2019 [6]	Retrospective	4,012	To investigate the role of routine HPE of appendectomy samples and its further impact on the management of patients	HPE confirms an unusual diagnosis which has an impact on patient’s management
Unver et al., 2019 [7]	Retrospective	2,047	To show the efficacy of HPE of appendectomy samples in making a diagnosis	Though small but significant (1.66%) number of unusual findings was seen suggesting routine HPE
Dincel et al., 2018 [8]	Retrospective	1,970	To find the frequency of unexpected pathologies in their hospital and compare the results	Unusual pathologies are often overlooked if routine HPE is not done
Omiyale & Adjepong, 2015 [9]	Retrospective	238	To correlate clinical and pathological diagnosis of acute appendicitis	Unusual pathologies usually impact the management of patient
Yabanoglu et al., 2014 [10]	Retrospective	1,466	To assess unusual pathologies by HPE of appendectomy specimen	Parasitic infection was the most frequently associated unusual pathology
Charfi et al., 2014 [11]	Retrospective	24,697	To analyze the necessity of routine HPE of appendectomy specimen	Though routine HPE is not cheap but still due to the probability of unusual diagnosis, routine HPE is recommended
Yilmaz et al., 2013 [12]	Retrospective	1,621	To understand the implications of unusual HPE findings in appendectomy patients	HPE helps in relatively early diagnosis and management in unusual cases
Emre et al., 2013 [13]	Retrospective	1,255	To analyze the benefit of routine HPE in appendicitis patients clinically	The probability of unusual findings is significant. Hence, routine HPE is recommended.
Chandrasegaram et al., 2012 [14]	Retrospective	4,670	To analyze various pathologies of appendix over a span of 10 years	Worm infection and fecaliths are one of the commonest usual causes of appendiceal colicky pain
Akbulut et al., 2011 [1]	Retrospective	5,262	To address the frequency of unusual findings in appendectomy samples	Though unusual findings are quite rare still it is beneficial to conduct routine HPE
Chamisa, 2009 [15]	Retrospective	371	To determine clinical presentations of acute appendicitis and review	Surgeons should always take into consideration parasitic infections and unusual causes as the reason for appendicitis
Lohsiriwat et al., 2009 [16]	Retrospective	4,545	To determine the impact of routine HPE of surgical specimens of appendix, hemorrhoids and gall bladder	Routine HPE of appendix and gall bladder specimens have shown significant benefits as compared to hemorrhoids
In’t Hof et al., 2008 [17]	Retrospective	1,485	To study the incidence and results of carcinoid tumors of the appendix	Carcinoids which are incidentally found usually have a good prognosis
Jones et al., 2007 [18]	Retrospective	1,225	To study HPE reports of appendectomy samples	Results from HPE had a significant impact on management and prognosis of the patient
Marudanayagam et al., 2006 [19]	Retrospective	2,660	To analyze the usual and unusual diagnosis of appendicitis	There are a significant amount of unusual pathologies, hence, favoring the policy of routine HPE.

Discussion

The appendix is a blind-ended tube that generally sits in the lower right quadrant of the abdomen. Earlier, the appendix has been considered as a rudimentary organ and labeled “vestigial organ” (organ which has lost its function along the evolutionary pathway) until recently when a significant number of studies have shown its immunoprotective role in the intestinal immune system [20].

Pathophysiology of Acute Appendicitis

The main underlying etiology of acute appendicitis is luminal obstruction of the appendix which may be due to lymphatic hyperplasia resulting in increased intraluminal pressure leading to edema, ulceration, and inflammation which also increases its susceptibility to bacterial infections. The usual causes of acute appendicitis include fecalith and lymphatic hyperplasia. The unusual causes of appendicitis can be - Infectious or Lesions (Benign/Malignant). The unusual infectious causes of appendicitis can be due to ascariasis, amebiasis, enterobiasis, actinomycosis, balantidiasis, tuberculosis, trichuriasis, or schistosomiasis [1]. Lesions such as benign neuroma, mucocele, mucinous cystadenoma, endometriosis, gastrointestinal stromal tumor, carcinoid tumors of the appendix, hyperplastic polyp, lymphoma, leukemia, granulomatous diseases, or leiomyoma can also be unusual causes of appendicitis [1].

Diagnosis and Management

Alvarado score is a clinical scoring system that takes into account six clinical features and two laboratory findings. It is a useful diagnostic score to rule out other diagnosis, however, its efficacy is inconsistent in children and overpredicts the chance of appendicitis in females. The score of 7-10 is highly suggestive and needs surgery. A score of 5-6 should not be discharged but kept in observation, however, a score of 1-4 can be discharged on the doctor’s discretion [21].

Table 2 depicts the scoring criteria of Alvarado score.

**Table 2:** Scoring criteria of Alvarado score

	Score Given	
	No	Yes
1. Clinical Signs		
1.a Right lower quadrant tenderness	0	+2
1.b Rebound pain	0	+1
1.c Increased temperature	0	+1
2. Symptoms		
2.a Anorexia	0	+1
2.b Nausea or vomiting	0	+1
2.c Migration of pain to the right lower quadrant	0	+1
3. Laboratory findings		
3.a Leukocytosis >10,000	0	+2
3.b Shift of WBC to left	0	+1

Role of Routine Histopathological Examination of Surgical Specimens

Routine HPE of a surgical specimen is generally considered beneficial as it helps to find out any unusual findings. However, due to the substantial costs of lab tests and limited resources available to the hospital, the efficacy and positive impact on the management needs to be significant to encourage routine pathological examinations.

A recent meta-analysis involving analysis of 4012 cases of acute appendicitis clearly stated that though the patients with unusual pathological findings are less (6.4%) it is still a significant value and impacts not only the prognosis but also the management of the patient. The whole treatment plan may need to be changed in case of unusual findings and therefore, clearly recommended routine HPE for all appendectomy specimens [6].

Another retrospective study involving systematic review and meta-analysis of 24,697 cases found the incidence of neoplastic lesions at 0.7% and unusual pathological diagnosis in 0.9%. Neoplastic lesions seen were carcinoid tumors, mucinous neoplasms, and adenocarcinoma. It concluded that though routine HPE is expensive but it is still recommended to continue doing routine histology because of a significant number of unusual diagnosis [11].

A clinical audit of a hospital in the United Kingdom on meta-analysis showed that about 1.7% of the total cases had unusual pathology other than inflammation of the appendix. These abnormal pathologies resulted in the change of the management plan of the patients. It justified doing routine HPE as there was a significant change in the management plan if unusual findings were seen [9].

To summarize, for a better holistic approach, several recent retrospective studies and meta-analysis have favored and recommended the policy of doing routine HPE of appendectomy specimens to rule out unusual pathologies.

Limitations

Even after all the accessible records available, our systematic review has some limitations. We only included literature available in the English language and excluded some abstracts for which we were unable to retrieve the full text. Non peer-reviewed articles were not included.

## Conclusions

Acute appendicitis is not caused only due to a single etiology but can occur due to a variety of causes which may be parasitic infections, benign or malignant lesions. On finding unusual etiology on histopathological examination of appendectomy specimen, it usually results in changing the management plan of the patient. There is sufficient evidence suggesting various uncommon etiologies that may present as acute appendicitis in several cases. Though lab costs may be substantial and unusual findings are only seldom seen but it is still recommended to perform a routine histopathological examination of all appendectomy specimens to rule out unusual pathologies.
